# Checklist of the family Pipunculidae (Diptera) of Finland

**DOI:** 10.3897/zookeys.441.7278

**Published:** 2014-09-19

**Authors:** Christian Kehlmaier

**Affiliations:** 1 c/o Senckenberg Natural History Collections Dresden, Museum of Zoology, Dresden, Germany

**Keywords:** Checklist, Finland, Diptera, Pipunculidae

## Abstract

A checklist of the Pipunculidae (Diptera) cited from Finland is presented. At present, 107 species have been recorded.

## Introduction

Pipunculidae or big-headed flies can be readily identified at family level by their large compound eyes occupying almost the entire globular head. Additional morphological autapomorphies at the family rank of this group are their enlarged anterior ommatidial facets in the female including a so far unique retina1 pattern among Diptera, the piercer-like shape of the female ovipositor and the presence of a chitinized postspiracular fig of their last instar larvae (Wada 1991, Rafael and De Meyer 1992). Due to the small to medium sized adults (2–12 mm), their uniform dark appearance and their rather cryptic lifestyle, it takes some effort to detect them in the field. Therefore, Pipunculidae can best be collected by Malaise traps, although a trained eye and patience can yield species rich catches by hand-netting as well. Big-headed flies can be found in a variety of habitats, where they are hovering in between herbal vegetation or among leaves of bushes and trees, searching for food sources (mainly honeydew) and mating partners or for suitable hosts for their parasitic larvae, which are important endoparasitoids of nymphal and adult Auchenorrhyncha (Chalarinae and Pipunculinae) and adult Tipulidae (Nephrocerinae) (Koenig and Young 2007). This highly specialized life-style is unique to this family of Diptera. Hardly any larvae have been described so far and the knowledge about their host specificity is fragmentary. For the European fauna, host records are available for 30% of the 209 known species, i.e., 62 pipunculid species have been reared from 59 host species in the past (Kehlmaier unpublished).

The knowledge of the Finnish fauna can be considered as good, with the latest review published only recently by Kehlmaier and Ståhls (2007). Whereas about a dozen additional species can be expected to be found, the distribution of the individual taxa within Finland is largely unknown.

**Identification:** Pipunculidae taxonomy largely depends on male genitalic features. Therefore, dissection of the male genital apparatus is essential and a high magnification of at least 50 times should be used for identification. Females can best be identified through a combination of outer anatomical features primarily based on the shape of the ovipositor. Most pipunculid genera present in Europe have been taxonomically reviewed within the past three decades. As no comprehensive key exists for northern Europe, adults have to be identified with the following set of publications: *Chalarus* (Kehlmaier and Assmann 2008, Kehlmaier 2010), *Jassidophaga* and *Verrallia* (Kuznetzov 1992, Kehlmaier 2006), *Nephrocerus* (Grootaert and De Meyer 1986), Cephalopsini (De Meyer 1989, Ackland 1993, Kehlmaier and De Meyer 2005, Kehlmaier 2008), Eudorylini (Kehlmaier 2005, Kuznetzov 1990), Pipunculini (Kehlmaier 2008), *Dorylomorpha* (Albrecht 1990), *Tomosvaryella* (Földvári and De Meyer 1999, Kehlmaier 2008).

**Number of species:**

World: 1428 species (Pape et al. 2011)

Europe: 209 species

Finland: 107 species

Faunistic knowledge level in Finland: good

## Checklist

suborder Brachycera Macquart, 1834

clade Eremoneura Lameere, 1906

clade Aschiza Becher, 1882

superfamily Syrphoidea Latreille, 1802

**PIPUNCULIDAE** Walker, 1834

CHALARINAE Aczél, 1939

***CHALARUS*** Walker, 1834

*Chalarus basalis* Loew, 1873

*Chalarus brevicaudis* Jervis, 1992

*Chalarus decorus* Jervis, 1992

*Chalarus elegantulus* Jervis, 1992

= *absconditus* Kehlmaier in Kehlmaier & Assmann, 2008

*Chalarus fimbriatus* Coe, 1966

*Chalarus gynocephalus* Jervis, 1992

*Chalarus holosericeus* (Meigen, 1824)

= *perplexus* Jervis, 1992

*Chalarus immanis* Kehlmaier, 2008

*Chalarus indistinctus* Jervis, 1992

*Chalarus juliae* Jervis, 1992

*Chalarus latifrons* Hardy, 1943

*Chalarus pughi* Coe, 1966

*Chalarus spurius* (Fallén, 1816)

= *obscurus* (Zetterstedt, 1838)

= *argenteus* misid.

***JASSIDOPHAGA*** Aczél, 1939

*Jassidophaga beatricis* (Coe, 1966)

*Jassidophaga fasciata* (von Roser, 1840)

= *setosa* (Verrall, 1901)

*Jassidophaga pilosa* (Zetterstedt, 1838)

*Jassidophaga villosa* (von Roser, 1840)

*Jassidophaga* spec. A

***VERRALLIA*** Mik, 1899

*Verrallia aucta* (Fallén, 1817)

NEPHROCERINAE Aczél, 1939

***NEPHROCERUS*** Zetterstedt, 1838

*Nephrocerus flavicornis* Zetterstedt, 1844

*Nephrocerus lapponicus* Zetterstedt, 1838

*Nephrocerus scutellatus* (Macquart, 1834)

PIPUNCULINAE Walker, 1834

tribe Cephalopsini Macquart, 1834

***CEPHALOPS*** Fallén, 1810

**sg. *Cephalops*** Fallén, 1810

*Cephalops aeneus* Fallén, 1810

*Cephalops vittipes* (Zetterstedt, 1844)

= *annulipes* (Zetterstedt, 1838) in part

**sg. *Parabeckerias*** De Meyer, 1994

*Cephalops obtusinervis* (Zetterstedt, 1844)

**sg. *Semicephalops*** De Meyer, 1994

*Cephalops carinatus* (Verrall, 1901)

*Cephalops straminipes* (Becker, 1900)

= *chlorionae* (Frey, 1945)

*Cephalops subultimus* Collin, 1956

*Cephalops varipes* (Meigen, 1824)

= *semifumosus* (Kowarz, 1887)

***CEPHALOSPHAERA*** Enderlein, 1936

*Cephalosphaera furcata* (Egger, 1860)

*Cephalosphaera germanica* Aczél, 1940

tribe Eudorylini Rafael & De Meyer, 1992

***CLISTOABDOMINALIS*** Skevington, 2001

*Clistoabdominalis doczkali* Kehlmaier, 2005

***EUDORYLAS*** Aczél, 1940

*Eudorylas angustimembranus* Kozánek & Kwon, 1991

= *kozaneki* De Meyer, 1993

*Eudorylas arcanus* Coe, 1966

*Eudorylas barkalovi* Kuznetzov, 1990

*Eudorylas carpathicus* Kozánek, 1993

*Eudorylas coloratus* (Becker, 1897)

*Eudorylas elephas* (Becker, 1897)

*Eudorylas fascipes* (Zetterstedt, 1844)

*Eudorylas furvulus* Collin, 1956

*Eudorylas fuscipes* (Zetterstedt, 1844)

= *roseri* misid.

= *trochanteratus* misid.

*Eudorylas fusculus* (Zetterstedt, 1844)

*Eudorylas goennersdorfensis* Dempewolf & Dunk, 1996

*Eudorylas inferus* Collin, 1956

*Eudorylas jenkinsoni* Coe, 1966

*Eudorylas johnenae* Dempewolf, 1996

*Eudorylas kowarzi* (Becker, 1897)

*Eudorylas montium* (Becker, 1897)

*Eudorylas obscurus* Coe, 1966

*Eudorylas restrictus* Coe, 1966

= *pannonicus* misid.

*Eudorylas slovacus* Kozánek, 1993

*Eudorylas stackelbergi* Kuznetzov, 1990

*Eudorylas subfascipes* Collin, 1956

*Eudorylas subterminalis* Collin, 1956

*Eudorylas terminalis* (Thomson, 1870)

*Eudorylas unicolor* (Zetterstedt, 1844)

*Eudorylas vonderdunki* Dempewolf, 1998

*Eudorylas zermattensis* (Becker, 1897)

*Eudorylas zonatus* (Zetterstedt, 1849)

*Eudorylas zonellus* Collin, 1956

tribe Microcephalopsini Rafael & De Meyer, 1991

***MICROCEPHALOPS*** De Meyer, 1989

*Microcephalops opacus* (Fallén, 1816)

= *vestitus* (Becker, 1900)

tribe Pipunculini Walker, 1834

***PIPUNCULUS*** Latreille, 1802

*Pipunculus calceatus* von Roser, 1840

*Pipunculus campestris* Latreille, 1802

= *ater* Meigen, 1824

= *spinipes* Meigen, 1830

= *thomsoni* Becker, 1897

*Pipunculus dimi* Kuznetzov, 1991

*Pipunculus elegans* Egger, 1860

= *spinipes* auct. nec Meigen, 1830

*Pipunculus fonsecai* Coe, 1966

*Pipunculus lenis* Kuznetsov, 1991

= *thomsoni* auct. nec Becker, 1897

*Pipunculus lichtwardti* Kozanek, 1981

*Pipunculus oldenbergi* Collin, 1956

*Pipunculus omissinervis* Becker, 1889

*Pipunculus tenuirostris* Kozanek, 1981

= *balticus* Kuznetzov, 1991

*Pipunculus violovitshi* Kuznetzov, 1991

= *varipes* auct. nec Meigen, 1824

*Pipunculus zugmayeriae* Kowarz, 1887

tribe Tomosvaryellini Hardy, 1943

***DORYLOMORPHA*** Aczél, 1939

**sg. *Dorylomorpha*** Aczél, 1939

*Dorylomorpha aczeli* (Hardy, 1947)

*Dorylomorpha confusa* (Verrall, 1901)

*Dorylomorpha extricata* (Collin, 1937)

*Dorylomorpha imparata* (Collin, 1937)

*Dorylomorpha rufipes* (Meigen, 1824)

= *xanthoceroides* (Aczél, 1939)

*Dorylomorpha spinosa* Albrecht, 1979

**sg. *Dorylomyia*** Albrecht, 1990

*Dorylomorpha beckeri* (Aczél, 1939)

*Dorylomorpha xanthocera* (Kowarz, 1887)

**sg. *Dorylomyza*** Albrecht, 1990

*Dorylomorpha albitarsis* (Zetterstedt, 1844)

*Dorylomorpha anderssoni* Albrecht, 1979

*Dorylomorpha canadensis* Hardy, 1943

*Dorylomorpha clavata* Albrecht, 1979

*Dorylomorpha clavifemora* Coe, 1966

*Dorylomorpha fennica* Albrecht, 1979

*Dorylomorpha hackmani* Albrecht, 1979

*Dorylomorpha haemorrhoidalis* (Zetterstedt, 1838)

*Dorylomorpha infirmata* (Collin, 1937)

*Dorylomorpha lautereri* Albrecht, 1990

*Dorylomorpha occidens* (Hardy, 1939)

*Dorylomorpha onegensis* Albrecht, 1990

*Dorylomorpha platystylis* Albrecht, 1979

*Dorylomorpha praetermissa* Albrecht, 1979

*Dorylomorpha xanthopus* (Thomson, 1870)

**sg. *Pipunculina*** Albrecht, 1990

*Dorylomorpha borealis* (Wahlgren, 1910)

*Dorylomorpha maculata* (Walker, 1834)

***TOMOSVARYELLA*** Aczél, 1939

= ***Alloneura*** Rondani, 1856 nomen nudum

*Tomosvaryella cilitarsis* (Strobl, 1910)

= *forsiusi* (Frey, 1932)

*Tomosvaryella coquilletti* (Kertész, 1907)

*Tomosvaryella geniculata* (Meigen, 1824)

= *nigritula* (Zetterstedt, 1844)

*Tomosvaryella kalevala* Kehlmaier, 2008

*Tomosvaryella kuthyi* Aczél, 1944

*Tomosvaryella minuscula* (Collin, 1956)

= *magyarica* Földvári & De Meyer, 1999

*Tomosvaryella palliditarsis* (Collin, 1931)

*Tomosvaryella rossica* Kuznetzov, 1993

*Tomosvaryella sylvatica* (Meigen, 1824)

## Excluded species (as discussed in Kehlmaier and Ståhls 2007)

*Claraeola halterata* (Meigen, 1838) misidentified

*Dorylomorpha incognita* (Verrall, 1901) misidentified

*Clistoabdominalis trochanteratus* (Becker, 1900) misidentified

*Dasydorylas roseri* (Becker, 1897) misidentified

*Eudorylas pannonicus* (Becker, 1897) misidentified

## Notes

***Chalarus argenteus* Coe, 1966**. The single Finnish specimen previously identified as *Chalarus argenteus* was re-examined and was found to belong to *Chalarus spurius* (Kaj Winqvist, pers. comm.).

***Jassidophaga* spec. A**. The specimens summarised under this place holder in Kehlmaier and Ståhls (2008) might represent an additional species. Using the key in Kuznetzov (1992), males run towards the Eastern Palaearctic *Jassidophaga kurilensis* (Kuznetzov, 1992), whereas females are closest to *Jassidophaga beatricis*.
